# Development of Machine Learning and Medical Enabled Multimodal for Segmentation and Classification of Brain Tumor Using MRI Images

**DOI:** 10.1155/2022/7797094

**Published:** 2022-08-24

**Authors:** L. Anand, Kantilal Pitambar Rane, Laxmi A. Bewoor, Jyoti L. Bangare, Jyoti Surve, Mutkule Prasad Raghunath, K. Sakthidasan Sankaran, Bernard Osei

**Affiliations:** ^1^Department of Networking and Communications, SRM Institute of Science and Technology, Chennai, India; ^2^Department of Electronics and Communication, Koneru Lakshmaiah Education Foundation (Deemed to Be University), Andhra Pradesh, Vaddeswaram, India; ^3^Department of Computer Engineering, Vishwakarma Institute of Information Technology, Savitribai Phule Pune University, Pune, India; ^4^Department of Computer Engineering, MKSSS's Cummins College of Engineering for Women, Savitribai Phule Pune University, Pune, India; ^5^Department of Information Technology, International Institute of Information Technology, Hinjewadi, Savitribai Phule Pune University, Pune, India; ^6^Department of Information Technology, SRES's Sanjivani College of Engineering, Kopargaon 423603, Maharashtra, India; ^7^Department of ECE, Hindustan Institute of Technology and Science, Chennai, India; ^8^Kwame Nkrumah University of Science and Technology, Kumasi, Ghana

## Abstract

The improper and excessive growth of brain cells may lead to the formation of a brain tumor. Brain tumors are the major cause of death from cancer. As a direct consequence of this, it is becoming more challenging to identify a treatment that is effective for a specific kind of brain tumor. The brain may be imaged in three dimensions using a standard MRI scan. Its primary function is to examine, identify, diagnose, and classify a variety of neurological conditions. Radiation therapy is employed in the treatment of tumors, and MRI segmentation is used to guide treatment. Because of this, we are able to assess whether or not a piece that was spotted by an MRI is a tumor. Using MRI scans, this study proposes a machine learning and medically assisted multimodal approach to segmenting and classifying brain tumors. MRI pictures contain noise. The geometric mean filter is utilized during picture preprocessing to facilitate the removal of noise. Fuzzy c-means algorithms are responsible for segmenting an image into smaller parts. The identification of a region of interest is facilitated by segmentation. The GLCM Grey-level co-occurrence matrix is utilized in order to carry out the process of dimension reduction. The GLCM algorithm is used to extract features from photographs. The photos are then categorized using various machine learning methods, including SVM, RBF, ANN, and AdaBoost. The performance of the SVM RBF algorithm is superior when it comes to the classification and detection of brain tumors.

## 1. Introduction

The improper and excessive growth of brain cells may lead to the formation of a brain tumor. Brain tumors are the major cause of death from cancer in those under the age of 19, accounting for 24 percent of all deaths caused by cancer. There are around 120 very different types of brain tumors. As a direct consequence of this, it is becoming more challenging to identify a treatment that is effective for a specific kind of brain tumor. For the purposes of categorization, brain tumors may be broken down into two primary subtypes: benign and malignant. Benign brain tumors are less dangerous than their malignant counterparts. When discussing the many kinds of cancer, the term “benign” refers to a tumor that does not metastasize, or spread, to other regions of the body. This kind of cancer is less likely to be fatal. Excision surgery is the standard treatment for benign tumors, which can be cured in most cases. Tumors that are cancerous have a high risk of spreading to other areas of the body and are notoriously difficult to remove [1, 2].

However, if the patient has any kind of brain tumor, whether it be malignant or benign, the patient is in danger and may even pass away. This is due to the fact that benign brain tumors may not invade the surrounding tissue, but as they get bigger, they place additional pressure on neighboring brain cells that are essential for normal brain function. This can cause brain damage. Brain tumors may originate in the tissue of the brain itself, or they might be brought there by cancer cells that have traveled to the brain from another part of the body. The process by which tumor cells from one source go to another organ and infiltrate the tissue of that new place is referred to as “metastasis,” and this is the meaning of the word “metastasis” [3, 4].

Using a regular MRI scan, a three-dimensional image of the human brain is possible to obtain. The major purpose of this organization is to investigate, recognize, diagnose, and categorize a wide range of neurological diseases. Radiation therapy is typically used in the treatment of cancers, and MRI segmentation is frequently utilized to direct radiation therapy. As a result of this, we are in the position to determine whether or not a fragment that was detected by an MRI is a tumor. These treatments do not involve the use of ionizing radiation and are instead used for diagnosis, the identification of disease stages, and subsequent monitoring. The most common setting in which these treatments are used is within hospitals. Magnetic resonance imaging (MRI), one of the most beneficial modalities that are presently available, is used to diagnose more advanced stages of brain tumors [5].

**Figure 1:**
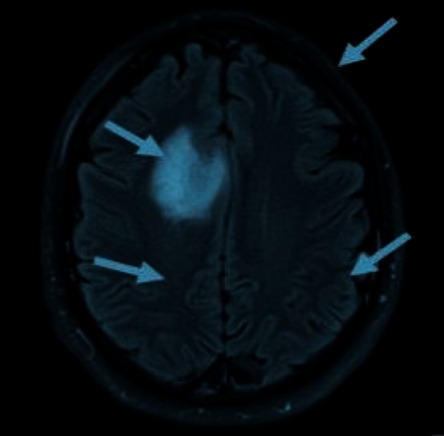
MRI image of brain tumor.

When looking for potentially hazardous areas in medical images, segmentation is an essential step that must be taken. There is potential for tremendous use and precision in the use of automated identification for the early diagnosis of brain tumors by MRI. The MRI of the brain tumor is shown in Figure 1.

The field of artificial intelligence encompasses a wide range of subfields, including machine learning and computational theory, searching and probability, as well as neuroscience [6]. To begin, it is fairly excellent at obtaining homogenous data from a big number of data, and it is able to learn from any form of data, whether it be numeric, visual, or video data. In addition, it can learn from any amount of data. Getting familiar with a dataset might provide you an advantage in the research and data extraction work you are doing. At the moment, those working in the field of medicine are having trouble diagnosing human diseases at an early stage so effective medicines may be created to assist patients in living longer lives. The identification of diabetic retinopathy, breast cancer, and lung cancer and the detection and diagnosis of brain tumors using MRI are some of the medical applications that make use of prescreening. It is very necessary to make use of image processing methods that facilitate automatic learning and the extraction of information. AI strategies have the potential to assist addressing a larger variety of complicated problems in a more timely and efficient manner [7, 8].

The literature review section contains a survey of existing techniques for brain tumor detection. The methodology section describes how to use MRI scans to segment and classify brain tumors using machine learning and medically enabled multimodal. Noise is seen in MRI pictures. The geometric mean filter is used in picture preprocessing to eliminate noise. FCM (fuzzy c-means) algorithms split images into parts. The identification of an area of interest is aided by segmentation. The GLCM Grey-level co-occurrence matrix is used to reduce the dimensions. GLCM is a program that extracts characteristics from photos. Finally, machine learning methods such as SVM RBF, ANN, and AdaBoost are used to classify pictures. The result section presents results achieved by various machine learning and feature extraction techniques. The conclusion section contains major contributions of the article to brain tumor detection.

## 2. Literature Survey

This section presents a literature survey of image preprocessing, feature extraction, segmentation, and classification techniques in the context of brain tumor detection.

### 2.1. Literature Survey of Image Preprocessing Techniques

The process of denoising MRI images was the focus of research that Agrawal and Sahu conducted [9]. Denoising an MRI picture was accomplished by the author via the use of the discrete wavelet transform (DWT). Wavelet analysis may be used to address discontinuities in higher derivatives, breakdown points, and other self-similarities that are overlooked by standard signal analysis approaches. It is able to denoise and compress a signal without compromising the signal's quality. The discrete wavelet transform not only creates an image that is free of unnecessary redundancies, but also provides additional spectral and spatial information. When compared to the information offered by the Gaussian or Laplacian pyramid, the information that it delivers is richer and more detailed. In the DWT method, low and high pass filters are responsible for the creation of approximation and detail coefficients. DWT may also be used to do the technique in reverse, which will restore the original signal in its entirety with no degradation.

An article on methods for improving medical image processing was authored by Bedi and Rati Khandelwal [10], and it was titled “Medical Image Processing Improvement Approaches.” The likes of medical pictures, aerial photography, and other sorts of imaging all suffer from issues with poor contrast and noisy signal. Increasing an image's contrast and sharpness and minimizing blurriness and making an image more obvious to the naked eye are some of the methods that may be used to improve picture quality. The spatial and frequency domain techniques are the two categories of strategies that may be used to enhance images. The quality of the image was enhanced by the use of spatial domain techniques including negative transforms, power-log transformations, piecewise slicing, and histograms. In order to produce smooth pictures in the frequency domain, Fourier transformations of an existing picture are used. These changes reduce the intensity of a predetermined range of frequency components.

Mathen and George are the ones who created a method for improving and denoising medical images [11]. The technique that was proposed consisted of three stages: preprocessing, enhancing the contrast, and denoising the image. The authors used the median filter in order to lessen the amount of noise and increase the level of clarity towards the margins. In the second phase, the histogram equalization method is used in order to increase the contrast of the image. To ensure that the grayscale values are distributed consistently over the picture, a preprocessing technique called as histogram equalization is used. The contrast of a picture that seems natural is improved by doing this. The histogram of a picture should be stretched in order to achieve the purpose of histogram equalization, which is to make the histogram as flat as possible. In an image of the brain taken with an MRI, it improves the contrast between the grey matter, the white matter, and the cerebrospinal fluid (CSF). At the very end of the procedure, the images were denoised by using a nonlocal means filter. It was able to reduce noise while maintaining the quality of the small details. The developer of the nonlocal means filter had the opinion that the image had a high degree of likeness to itself in the context of this situation.

### 2.2. Literature Survey of Image Segmentation Techniques

Dina et al. [12] demonstrated how a modified image segmentation approach might be used to analyze MR images for the purpose of locating brain tumors. Their learning vector quantization (LVQ)-based modified probabilistic neural network (PNN) model surpassed all others in training, classification with a hundred percent accuracy, and a decrease in compute time of seventy-nine percent in the processing of images and data. It has been shown and researched how linear and Gaussian filters work, in addition to techniques of augmentation and smoothing. The Canny edge detection technique was also used in the research on edge detection at various points.

Leela and Veenakumari [13] developed a computer-aided detection method for aberrant tissue growth with the purpose of improving medical diagnosis. This method involves the processing of MR images with the highest possible accuracy and speed. Additionally, they have improved the image quality by cutting down on the amount of noise that was there. The images of brain tumors, as well as morphological processing and segmentation procedures, have all been explored for their qualities of discontinuous or comparable intensity values.

In their 2014 study, Rohini Paul et al. [14] suggested using the K-means clustering approach to separate the data obtained from brain MRI scans. In order to prevent the formation of clustered areas during the segmentation of brain MRI images for the purpose of diagnosing tumors, morphological filtering is an absolute need.

This is the first time that Hamoud et al. have offered [15] a complete evaluation of the technique and methods that were used. Motifs and edges wrap up with a perceptive summary that paves the way for further study on brain picture segmentation, thresholding, and noise reduction, as well as cancer diagnostics.

Using photographs of brain tumors, Siva Sankari et al. developed a technique for segmenting the images and extracting information from them [16]. The K-means clustering method was used so that this could be accomplished. Reddy et al. [17] have devised a method for estimating the volume of a tumor by making use of the data from multi-parametric MR images, in addition to coming up with the novel concept of a confidence surface to assist in segmentation. In order to train the classifier and the segmentation approach, contrast-weighted image information and texture information were both used.

Using quantitative methodologies for automated feature identification based on the identity of each pixel in the picture, Megha et al. [18] demonstrated how to improve visual discrimination between scene features and replace visual analysis of image data with quantitative methods. Additionally, they demonstrated how to increase visual discrimination between scene features. Extraction of data via the use of digital image processing has also been considered.

Researchers Hemlatha et al. [19] discovered that it was possible to automatically establish the location of a tumor in the brain as well as its size by using MRI technology. They have shown how to do this via the use of digital picture processing.

### 2.3. Literature Survey of Feature Extraction Techniques

Mohanaiah et al. proposed a technique for image processing that retrieves textural information from a picture [20]. The Grey-level co-occurrence matrix (GLCM) approach was used in order to extract textural properties. Image analysis would not be complete without the use of texture analysis in the picture-sorting process. The GLCM algorithm may be used to extract textural properties from grayscale photographs. Ailments may be categorized as either normal or abnormal by applying these factors in the software used in medicine. This statistical method allows for the extraction of features. The author used this matrix technique in order to evaluate the properties of image movements. In order to evaluate properties such as angular second moment (energy), correlation, and entropy, an MRI image was used. This approach is more efficient in terms of time consumption when compared to the discrete wavelet transform.

In an article, Ping Tian and colleagues [21] proposed a method for extracting color image attributes. This method was included in the work. In RGB color photographs, a pixel's color is considered a property, but in greyscale photos, the texture is considered a property of an entire group of pixels. Images and the domains they belong to are used in order to obtain the statistical or pixel structure of a texture. This study looks at two different kinds of characteristics: local and global. Both of these are analyzed in this research. The LBP, Gabor wavelet, and histogram methods are used in this study to extract the characteristics of color images. The histogram method may be used in order to extract global features from an image. On the other hand, the Gabor wavelet and the Sobel shape detector can be utilized in order to extract local features from an image. When making the image portrayal, these considerations were taken into account; thus, the result is accurate.

An algorithm for the identification of brain tumors was suggested by Kourosh Jafari [22]. This approach makes use of high-resolution (HR) images with various degrees of contrast. The majority of the time, these low-contrast photographs are upsampled with high-contrast images to produce higher resolution versions of the original photos. The algorithm that is being presented uses an approach that is based on patches. The intensity of one pixel is compared to the intensity of all of the other pixels in the image to generate a similarity map using this approach. In this particular investigation, the authors obtained edge information by using a Gaussian filter.

### 2.4. Literature Survey of Classification Techniques

Unsupervised learning-based neural networks were proposed by Goswami and Bhaiya [23] for the categorization of brain tumors. The first step of the brain tumor diagnosis technique is picture preprocessing, and the second phase is the extraction of tumors from MR images. Equalization of histograms, edge detection, noise filtering, and thresholding were all used in the picture preprocessing procedure. Independent component analysis was used to extract the brain feature (ICA). The self-organized map is used in the third phase to diagnose brain cancers (SOM). The brain scans were segmented using the K-means technique. Classification based on unsupervised learning was shown to be promising in the segmentation of brain tumor pictures in the aforementioned investigation.

Brain cancers may be detected using an automated approach [24]. A multi-stage tumor extraction procedure was used to identify the brain tumor automatically. The MR scans of the brain have the noise reduced. Features were then retrieved from noise-free brain scans, as seen in the figure. It was based on the extracted characteristic that classified the brain tumors. Brain tumors were classified using ensemble-based SVM. With the SVM-based classification approach, it was able to attain a 99 percent accuracy rate. The classification method uses multi-step segmentation techniques to identify the tumor in the brain MR images. For example, after removing the skull, FCM clustering methods were used to retrieve the afflicted region's brain tumors for inclusion in the algorithm.

The SOM clustering provided by Vaishnavee and Amshakala [25] was used to segment the brain pictures. Before the pictures are segmented, histogram equalization is used to extract the features. For the selection of features and to increase the accuracy of the classifiers, principal component analysis (PCA) was utilized. Proximal support vector machines (PSVM) classifiers were also developed, which were more successful than the SVM classifiers. According to our findings, the SVM classifier is a powerful classifier for extracting features from digital photos and other visual data.

## 3. Methodology

**Figure 2:**
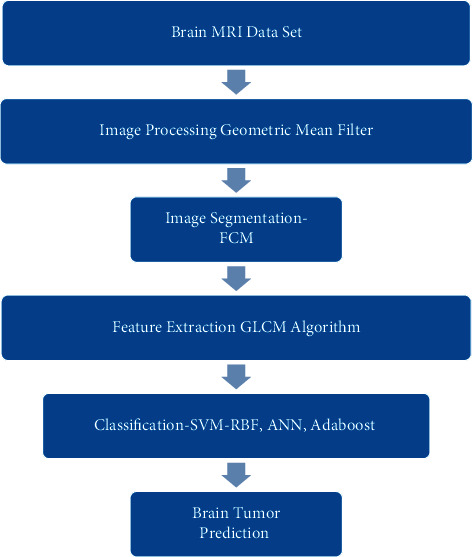
Machine learning and medically enabled multimodal for segmentation and classification of brain tumor using MRI images.

Using MRI images, this part presents machine learning and medically enabled multimodal for the segmentation and classification of brain tumors. MRI pictures contain noise. The geometric mean filter is utilized during picture preprocessing to facilitate the removal of noise. Fuzzy c-means algorithms are responsible for segmenting an image into smaller parts. The identification of a region of interest is facilitated by segmentation. The GLCM is utilized in order to carry out the process of dimension reduction. The GLCM algorithm is used to extract features from photographs. The photos are then categorized using various machine learning methods, including SVM, RBF, ANN, and AdaBoost. This model is shown in Figure 2.

Image preprocessing makes it feasible for illnesses seen in photographs to be classified in a more specific manner. Noise is the most prevalent type of picture artefact that can be observed in MRI scans; nevertheless, there is a broad range of image artefacts that can be noticed. Utilizing a number of distinct image filtering methods makes it possible to eliminate these artefacts from the image. In order to reduce the amount of noise present in the input photographs, a filter based on the geometric mean is applied to each of the images [26].

Clustering is a method that groups together patterns that are similar in order to discover the underlying connections that exist between individual pixels in an image. The process of putting things into groups or clusters based on the characteristics they have in common is what the term “clustering” refers to. When using the FCM methodology, the data items are sorted into groups according to the membership values of each category. Before splitting the final data, it is crucial to use the least squares strategy in order to maximize the effectiveness of the object function [27].

Using a method known as feature extraction, which is part of the field of image processing, it is possible to exclude dimensions from a collection of feature subsets that are thought of as being unnecessary or unimportant. When texture properties need to be restored and links between pixels need to be maintained, the GLCM technique is called for. One way to go about doing this is by computing the co-occurrence values of the various grey levels. In order to construct the general linear model (GLM), conditional probability density functions known as *pIj*|*d* and ş are utilized. After the GLM has been constructed, it is tested using distances *d* that range from one to five times the given direction (*ş* = 0, 45, 90, or 135). In order to accomplish this goal, the GLCM algorithm is used. According to the illustration, the probability that two pixels with the same grey level (*I* and/or *j*) as well as the inter-sample distance (*d* and ș) are spatially connected may be determined by applying the formula *p*(*i*, *j*|*d*, and ș). This formula also provides information about the distance between the samples (*d*). Contrast, correlation, entropy, and homogeneity are some of the most essential features of the general linear correspondence model (GLCM) [28].

This method, which is also known as AdaBoost, may be implemented to increase the accuracy of classification results produced by classifiers that are not all that great. This technique, known as AdaBoost, is used in the process of allocating initial weights to each observation. After a few iterations, incorrect categorizations will be given greater weight, while proper categorizations will be given less weight as the results are iterated over. The weights that are given to each observation are determined by the classification to which it belongs. This was done so that the performance of the classifier might be improved. Because of this, the possibility of incorrect categorization is reduced. A large number of kids who are having difficulty academically are progressively fitted in an adjustable fashion using the process known as “boosting.” In each consecutive model [29] in the series of models, there is a greater emphasis on information that had been ignored in earlier models.

In the realm of medicine, artificial neural networks (ANNs) are often used for the purpose of classifying medical images in order to arrive at a diagnosis. In many respects, including the manner in which it performs its duties, the ANN is comparable to the human brain. It is feasible to get the information required to make an informed guess about the category that an image belongs to by looking at a collection of photos that have already been categorized. This may be accomplished by looking at a collection of photographs that have been categorized. This may be accomplished by searching through a database of photos that have been arranged into a variety of different classifications. Every picture in this collection belongs to one of the categories that are listed above. ANNs are constructed by artificial neurons, each of which is programmed to perform in a manner that is analogous to the biological neurons found in the human brain. Connections allow for communication to take place between neurons that are located outside of the body. During the course of the learning process, weights may be given to neurons and edges, and those weights can be changed at any time according to the requirements of the task at hand. The standard structure of an artificial neural network consists of three layers: an input layer, a hidden layer, and an output layer that is responsible for signal generation. The majority of buildings will have this particular architectural style. Although the most typical topologies for artificial neural networks consist of input, hidden, and final layers, it is possible to configure the network in a variety of other ways. In theory, there may just be one hidden layer, there may be many hidden levels, or there may not be any hidden levels at all. It is within one's power to bring any one of these possibilities into fruition and make it a reality. In the event that it is required, the weights on a lower layer may be adjusted until the desired outcome is attained [30].

When it comes to the design of symbols, support vector machines provide a method that can discern the difference between the conceptions of measurement held by youngsters and those held by adults [31]. The following is a concise summary of the grouping issues faced by SVMs: (a) a nondirect translation of the information space to the higher measuring gimmick space, and (b) the construction of the distinguishing hyperplane that has the greatest distance from the purposes that are most closely related to the training set. Due to the direct distinct information, the SVM works toward locating, among all hyperplanes that minimize the preparation lapse, the particular case that distinguishes the preparation information with the greatest extreme separation from their nearest points of separation. This is done in an effort to minimize the amount of time that is lost during the preparation process.

## 4. Result Analysis and Discussion

For the purpose of this investigation, one hundred pictures were chosen at random from Ref. [32]. Only 25 of the photos have tumors, while the other 75 are healthy and unaffected. In order to train the model, 80 photographs are used, and then 20 of those images are used for testing. The geometric mean filter is utilized during picture preprocessing to facilitate the removal of noise. Fuzzy c-means algorithms are responsible for segmenting an image into smaller parts. The identification of a region of interest is facilitated by segmentation. The GLCM is utilized in order to carry out the process of dimension reduction. The GLCM algorithm is used to extract features from photographs. The photos are then categorized using various machine learning methods, including SVM, RBF, ANN, and AdaBoost.

**Figure 3:**
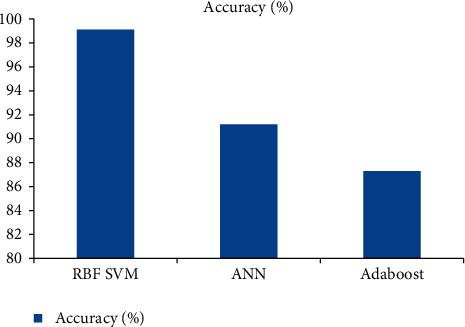
Accuracy comparison of classifiers for brain tumor detection.

**Figure 4:**
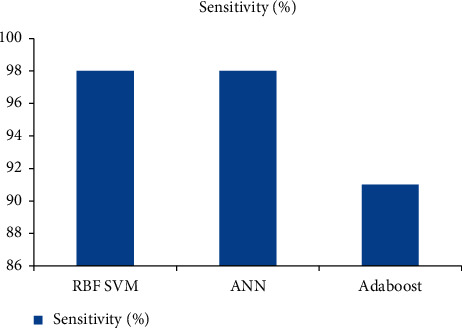
Sensitivity comparison of classifiers for brain tumor detection.

**Figure 5:**
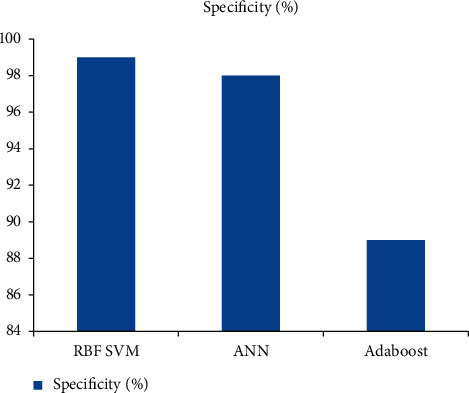
Specificity comparison of classifiers for brain tumor detection.

Within the scope of this investigation, a wide variety of algorithmic approaches are dissected and contrasted in terms of the degrees to which they exhibit accuracy, sensitivity, and specificity. Figures 3, 4, and 5 present the results of the classification performed by the algorithm. When it comes to the classification of brain tumors, the SVM RBF algorithm possesses an unrivaled level of accuracy and specificity that cannot be matched by any other method.

## 5. Conclusion

The abnormal and uncontrolled multiplication of brain cells may sometimes result in the development of a brain tumor. The majority of people who pass away from cancer do so because of brain tumors. As a direct result of this, it is becoming more difficult to pinpoint a therapy that is beneficial for a particular kind of brain tumor. Using a regular MRI scan, a three-dimensional picture of the human brain is possible to get. The major purpose of this organization is to investigate, recognize, diagnose, and categorize a wide range of neurological diseases. Radiation therapy is often used in the treatment of cancers, and MRI segmentation is frequently used in order to direct radiation therapy. As a result of this, we are in a position to determine whether or not a fragment that was detected by an MRI is a tumor. Using MRI scans, this study offers a machine learning and medically enabled multimodal for segmentation and classification of brain tumors. MRI images contain noise. The geometric mean filter is used during picture preprocessing to facilitate the removal of noise. Fuzzy c-means algorithms are responsible for segmenting an image into smaller parts. The identification of an area of interest is facilitated by segmentation. The GLCM is used in order to carry out the process of dimension reduction. The GLCM algorithm is used to extract features from photographs. The photos are then categorized using several machine learning methods, including SVM, RBF, ANN, and AdaBoost. VM with RBF kernel is better for brain tumor detection using MRI images.

## Data Availability

The data shall be made available on request.
